# Supplementary material for: Essential Oil Chemotypes and Genetic Variability of Cinnamomum verum Leaf Samples Commercialized and Cultivated in the Amazon
Source: Molecules. 2022 Oct 28;27(21):7337. doi: 10.3390/molecules27217337 (PMC9655072; doi:10.3390/molecules27217337)
Supplement: Supplementary file 1 [file molecules-27-07337-s001.zip › molecules-1869619 -Supplementary Materials.pdf]

Supplementary Material

Essential oil chemotypes and genetic variability of *Cinnamomum verum* leaf samples commercialized and cultivated in the Amazon

Júlia Karla A. M. Xavier [1], Talissa Gabriele C. Baia[2], Oscar Victor C. Alegria[3], Pablo Luis B. Figueiredo[4], Adriana R. Carneiro[3], Edith Cibelle de O. Moreira[5], José Guilherme S. Maia[1,6],William N. Setzer[7], and Joyce Kelly R. da Silva[1,7]*

[1] Programa de Pós-Graduação em Química, Universidade Federal do Pará, 66075-900, Belém, Brazil; julia.xavier@icen.ufpa.br (J.K.A.M.X)
[2] Programa Institucional de Bolsas de Iniciação Científica, Universidade Federal do Pará, 66075-900, Belém, Brazil; talissacaldasb@gmail.com (T.G.C.B.)
[3] Centro de Genômica e Biologia de Sistemas, Universidade Federal do Pará, 66075-900, Belém, Brazil; oscar.alegria@icb.ufpa.br (O.V.C.A); adrianarc@ufpa.br (A.R.C.)
[4] Departamento de Ciências Naturais, Centro de Ciências Sociais e Educação, Universidade do Estado do Pará, Belém, PA 66050-540, Brazil. pablo.figueiredo@uepa.br (P.L.B.F.)
[5] Instituto de Estudos em Saúde e Biológicas, Universidade Federal do Sul e Sudeste do Pará, 68501-970, Marabá, Brazil. cibelle@unifesspa.edu.br (E.C.M);
[6] Programa de Pós-Graduação em Química, Universidade Federal do Maranhão, São Luís, MA 65080-805, Brazil; gmaia@ufpa.br (J.G.S.M.)
[7] Aromatic Plant Research Center, 230 N 1200 E, Suite 100, Lehi, UT 84043, USA. wsetzer@aromaticplant.org (W.N.S.)
* Correspondence: joycekellys@ufpa.br (J.K.R.S.)

Table S1. Pairwise analysis of the *psbA-trnH* region using Kimura two-parameter method.

	Cve1	Cve2	Cve3	Cve4	Cve5	Cve6-c	Cve7-c	Cve8-c	Cve9-c
**Cve1**									
**Cve2**	0,014								
**Cve3**	0,007	0,012							
**Cve4**	0,002	0,017	0,010						
**Cve5**	0,010	0,014	0,002	0,012					
**Cve6-c**	0,010	0,014	0,002	0,012	0,000				
**Cve7-c**	0,017	0,027	0,015	0,019	0,012	0,012			
**Cve8-c**	0,002	0,017	0,010	0,005	0,012	0,012	0,019		
**Cve9-c**	0,019	0,029	0,027	0,017	0,029	0,029	0,037	0,022	

C. verum cultivated (**Cve1**-**Cve5**); *C. verum* commercials (**Cve6-c**-**Cve9-c**).
